# A video-based real-time adaptive vehicle-counting system for urban roads

**DOI:** 10.1371/journal.pone.0186098

**Published:** 2017-11-14

**Authors:** Fei Liu, Zhiyuan Zeng, Rong Jiang

**Affiliations:** 1 School of Hydropower and Information Engineering, Huazhong University of Science and Technology, Wuhan, China; 2 Huawei Corporation, Shenzhen, China; Chongqing University, CHINA

## Abstract

In developing nations, many expanding cities are facing challenges that result from the overwhelming numbers of people and vehicles. Collecting real-time, reliable and precise traffic flow information is crucial for urban traffic management. The main purpose of this paper is to develop an adaptive model that can assess the real-time vehicle counts on urban roads using computer vision technologies. This paper proposes an automatic real-time background update algorithm for vehicle detection and an adaptive pattern for vehicle counting based on the virtual loop and detection line methods. In addition, a new robust detection method is introduced to monitor the real-time traffic congestion state of road section. A prototype system has been developed and installed on an urban road for testing. The results show that the system is robust, with a real-time counting accuracy exceeding 99% in most field scenarios.

## Introduction

Many large cities in China are suffering from severe traffic problems as their vehicle populations have become unprecedentedly large. In addition to updating road networks, local governments are developing their own intelligent transportation systems (ITSs) to address the new challenges of traffic crowding. One of the basic components of an ITS is the vehicle counting system, which is designed to collect traffic flow information. In this paper, we focus on developing a vehicle counting system to be installed in urban bridges and tunnels. The basic requirements for the new system are as follows: (a) its counting accuracy must be higher than 98%; (b) it must be robust in various weather and light conditions, and it should work well at night; (c) it must be able to work online continuously for 24 hours a day to count vehicles at any time and at any site; and (d) the system maintenance should not consume substantial resources.

There are many ways to detect and count the vehicles driving along a specific road during a specific time period [1]. Some cities have implemented vehicle counting by deploying inductive loops. These loops provide high precision but are very intrusive to the road and come with a high maintenance cost [2–4]. Other devices, such as pyroelectric infrared sensors or ultrasonic detectors, make vehicle detection suitable for nighttime scenarios and can obtain more detailed information about the vehicles, such as width, length, and speed [5–6]. Some other papers provide qualitative behavior of adaptive vehicle-counting systems [7–11]. However, these methods are sensitive to noise and weather conditions, making them insufficiently robust.

In recent years, with the development of advanced computer infrastructure and digital image processing, the use of video-based technologies in vehicle detection has received increasing attention. Video sources can provide overall information about the vehicles and are easy and cheap to both obtain and maintain; thus, video-based technologies are mainstream methods in vehicle detection tasks.

Saad M. AI-Garn and Adel A. Abdennour adopt an automatic background updating algorithm and incorporate edge detection methods for background subtraction, obtaining a detection accuracy of nearly 91% [12]. However, the accuracy of conventional methods can also reach that level. Robert divides vehicle detection into daytime and nighttime detection processes to address the different background illumination conditions [13]. Improving the robustness of the system is a good start, but it still fails to consider the specifics of traffic jams, which would require new technologies, such as a feature-based detection method [14]. Watanabe et al. [15] construct a system based on machine learning, which employs genetic algorithm and edge detection methods [16]. The complicated methods show promising results but consume considerable resources, and ensuring their real-time performance is difficult. Many other systems, most of which can be derived or expanded from existing systems, focus on vehicle detection [17–21].

The existing methods have the following deficiencies: (a) their accuracy levels are relatively low; (b) their detecting and counting methods have not focused on traffic jams, leading to low robustness, and their performance in bad weather conditions cannot meet the standards; (c) many of these methods seek to improve the detection accuracy by sacrificing the real-time performance to some degree; and (d) most of these methods are designed for a common environment.

To overcome the challenges described above, we constructed an integrated real-time vehicle counting system with the following concepts: a) a block-wise background update mechanism to reduce the amount of calculation required and to improve the vehicle detection efficiency; b) separate algorithms for day and night and for free and congested traffic flows to improve the robustness of the system; and c) virtual loop, virtual line detection and adaptive vehicle counting methods for distinct traffic occasions to achieve precise real-time counting. The outcome system was run in a long-term test, and the test results were collected for validation. The results indicate that the counting accuracy reaches 99% in most of the tested scenarios. In addition, the program runs all the time, takes less than 70 ms to process a frame and can handle most practical scenarios robustly.

This paper is organized as follows. The overall structure of the system is described in Chapter II. The main methods of vehicle detection are detailed in Chapter III. Chapter IV presents the novel traffic congestion detection technique. In Chapter V, the adaptive vehicle counting method is introduced. The results and discussions are presented in Chapter VI. We conclude this paper in Chapter VII.

## Overview of the model

**Fig 1:**
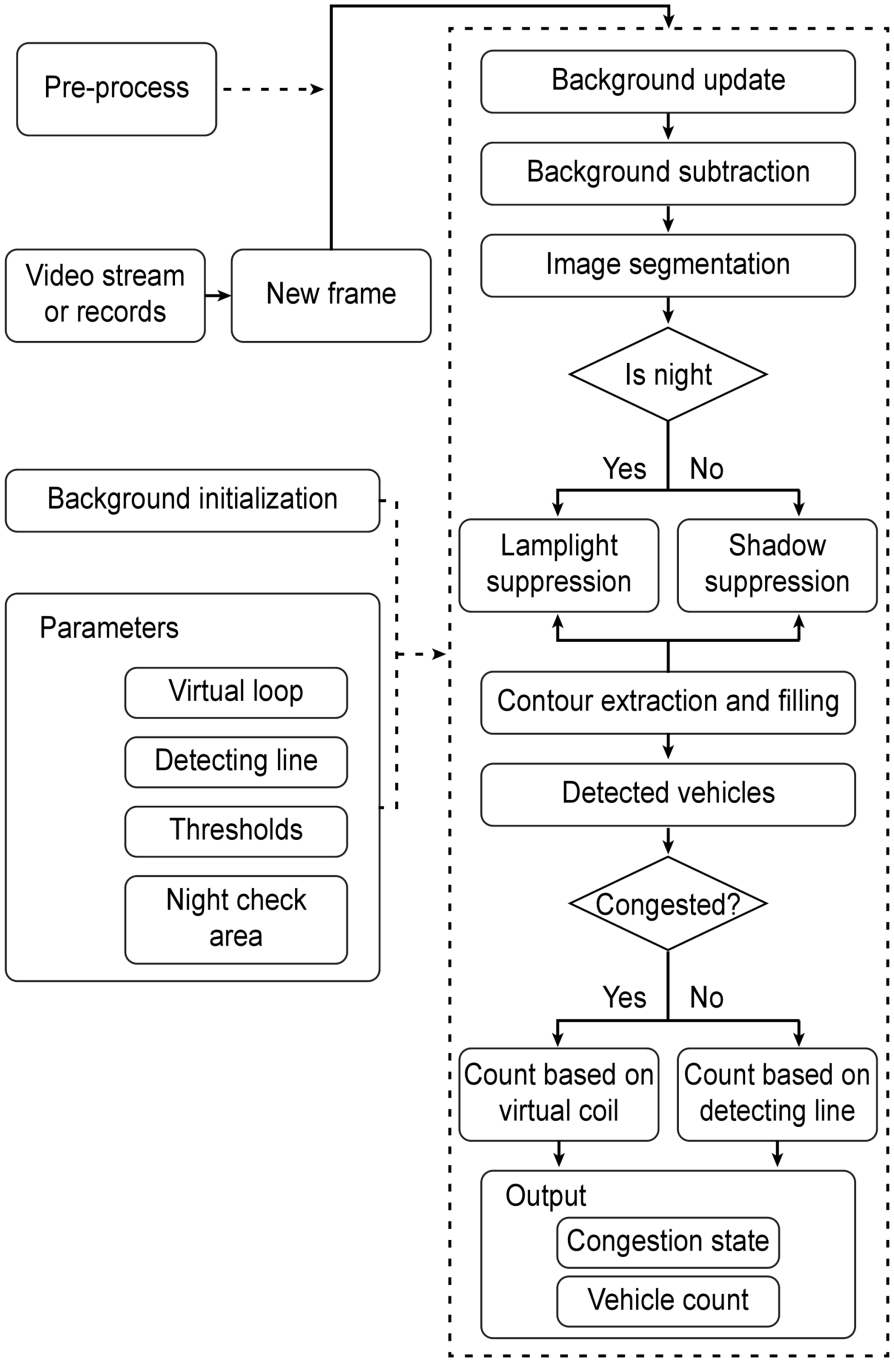
The flow chart of the system.

The flow chart for the vehicle detection and counting algorithm is shown in Fig 1. The main configuration parameters, such as virtual coil generation, line detection parameters, threshold values and night detection area, are predefined manually. The program begins with background initialization, which paves the way for subsequent processes. When a new frame is pushed into the system, the congestion status (congested or free) is determined using the method described in Chapter IV. Two different paths are then designed, one for congested traffic and one for free traffic. In one path, virtual coils are generated to count the vehicles in congested traffic, which are relatively slow. In the second path, a technique based on virtual line detection records the number of vehicles in free traffic, which are relatively fast. Before the vehicles are formally detected, some required operations are executed in order, such as fundamental image filtering, background subtraction, image segmentation, lamplight suppression (for nighttime conditions), shadow suppression (for daytime conditions), contour extraction and filling. When all the steps have been executed, the newly detected vehicles are counted and stored along with the traffic congestion status.

## Vehicle detection method

### Background update

Background subtraction is the most common method for identifying moving objects and is adopted in this paper. Background modelling methods are categorized into non-recursive and recursive method. Non-recursive methods, for example, frame difference, are highly adaptive because they use history frames as references but are not sensitive to slowly changing light conditions. Recursive methods, which continuously update the background to produce more adequate results, add strict demands to the anterior background because any error in the initial model will linger for a long period. Many mathematical models and techniques, such as the mixture Gaussian model, Kalman filter, optical flow and code book, have been adopted to mitigate background subtraction problems. However, complicated models always come with a trade-off. Most of the models require well-designed initial data or proper training processes. The time performance of these models is not comparable to that of non-parametric models. In this paper, we propose a block-wise background updating mechanism that recursively updates the background using simple equations based on the statistics determined from history frames. The basis of this method is described below.

#### An indicator of background update

The core idea of our background update mechanism is that when a pixel **p** is pure background or is covered by targets for a certain period of time, the recursive background update should be suspended. The first step is to find an indicator to determine when to update the background.

For any pixel **p** in a series of consecutive frames I_1_, I_2_, …I_N_ we assume that the corresponding values are *i*_1,**p**_, *i*_2,**p**_, *… i*_N,**p**_. Assuming a uniform load surface and uniform target shells, the gray level of the pixel can be simulated using the following equation:

$$i_{n,\text{p}}=\beta_{n,\text{p}}B$$ (1)

Here, B is the pixel value of the pure background, and *β* is the ratio between the pixel value of the target and the background.

$$\beta_{n,\text{p}}=\left\{ \begin{array}{l} \beta ,\text{when}\;\text{p}\;\text{is}\;\text{covered}\;\text{by}\;\text{objects}\\ 1,\text{otheriwse} \end{array}\right.$$ (2)

If there are N^’^ frames in the frame series where **p** is occupied by targets, we can obtain the pixel expectation:

$$E=\frac{N^{\prime }}{N}\beta B$$ (3)

Let *η* = N^’^ /N. The standard deviation is

$$\text{Std}(\text{p})=\sqrt{E(\beta_{n,\text{p}}B-\frac{N^{\prime }}{N}B)}=\sqrt{\eta (1-\eta )}\beta B$$ (4)

Define f(*η*) as Eq (5):

$$f(\eta )=\sqrt{\eta (1-\eta )}$$ (5)

f(*η*) is an inverted parabolic that achieves its extreme value 0.5 when *η* = 0.5 and *β*B is the pixel value of the targets. As can be concluded from Eq (4), when *η* approaches 0 or 1, Std(**p**) approaches 0 with dramatic speed, which indicates that **p** and its neighborhood are always pure background or pure targets in the specified frame series. Therefore, it is not necessary to update the background model in **p** or its neighborhood. When *η* is near 0.5, Std(**p**) is a perfect approximation of the target pixel value *β*B, which indicates that the pixel and its neighborhood are covered by moving targets. Thus, we choose Std(**p**) as the indicator of the block **P** that is centered at **p** to determine whether to update the background model of **P**.

#### The management of ‘ghosts’

A common problem of most traditional background subtraction algorithms is the generation of ghost artifacts. The commonly used equation for background updating is

$$B_{n,\text{p}}=(1-\alpha )B_{n-1,\text{p}}+\alpha I_{n,\text{p}}={\left(1-\alpha \right)}^{n}B_{0,\text{p}}+\alpha \sum_{i=0}^{n}{\left(1-\alpha \right)}^{n-i}I_{i,\text{p}}$$ (6)

When n is sufficiently large, (1-*α*)^*n*^B_0,**p**_ approaches 0, which indicates that the initial background matters little in the background update process.

All of the frames when **p** is covered by target objects constitute a frame set **T** whose length is t. Then, I_*i*,**p**_ is expressed by

$$I_{i,\text{p}}=\left\{ \begin{array}{l} \beta \text{B},i\in \text{T}\\ \text{B},\text{otherwise} \end{array}\right.$$ (7)

Then, we calculate the logarithm of the second item of Eq (6):

$$\text{log}\sum_{i=0}^{n}{\left(1-\alpha \right)}^{n-i}I_{i,\text{p}}=\text{log}B+\text{log}(\sum_{i\in T}{\left(1-\alpha \right)}^{n-i}\beta +\sum_{i\in N-T}{\left(1-\alpha \right)}^{n-i})$$ (8)

We assume that there are *k* uniform vehicles that pass pixel **p** at the same speed. The vehicles can be represented by V = {v_1_, v_2_, …, v_*k*_}. Each vehicle passed **p** in *l* frames. Vehicle v_*k*_ first touched pixel **p** in frame *n*(v_*k*_) and left **p** in frame *n*(v_*k*_)+*l*. N contains all the frames in the computation range L whose length is commonly set to be 200. Typically, when *α* = 0.1, (1-*α*)^200^ is sufficiently small to be neglected. Calculate the two items of Eq (8):

$$\rho (n)={\sum_{i\in T}\left(1-\alpha \right)}^{L-i}=\theta^{\text{L}-l}\sum \theta^{-n(v_{k})}(1-\theta^{l})/(1-\theta )$$ (9)

$${\sum_{i\in N-T}\left(1-\alpha \right)}^{L-i}=(1-\theta^{L})/(1-\theta )-\rho (n)$$ (10)

Where *θ* = 1-*α*. After deduction, B_*n*,**p**_ can be obtained:

$$B_{n,\text{p}}\approx B+[\alpha \rho (n)(\beta -1)B]$$ (11)

As shown in the above equations, the item in the bracket cause ghost artifacts. It is easy to find that when no vehicles appear in the calculation range *ρ*(*n*) = 0 which makes B_*n*,**p**_ equals to the real background B. To restore B from B_*n*,**p**_, the value of the formula in brackets should be determined. Technically, *ρ*(*n*) can be calculated by recording the frames when target objects first touch pixel **p** and the frame when the object leaves **p** (to calculate the length *l*).

To obtain the indicated time, we define a calculation window for block *i*:

$$\left(\{n_{v(k)}\},\beta )={\text{CW}}_{i,L}(a_{i},f_{i},th1,th2\right)$$ (12)

The length of CW is L. By shifting CW_*i*,L_ from n to 1, the standard deviation is *a*_i_ and the average pixel *f*_*i*_ are calculated. *th*1 and *th*2 are the specified threshold values. When *a*_i_ <*th*1, CW is covering the time range of pure objects or pure background; otherwise the state of the pixel is changing. If the current background model is b_*i*_, then the value of |*f*_*i*_* − b*_*i*_| can be used to determine whether the pixel is covered by objects. We can obtain the n_v(*k*)_ by recording the frame that object v(*k*) first touch pixel **p**. For every frame, if |*f*_*i*_* − b*_*i*_| > *th*2, then we record its value. We can thus calculate the average foreground pixel *f*_0_. Then *β* can be expressed by:

$$\beta =f_{0}/b$$ (13)

Inevitable errors may occur in the process of removing ghost artifacts. Therefore, we apply the adaptive local noise reduction filter based on the local statistics of the image.

#### The computational procedure

1. First, partition the ROI (region of interest) of the target frame into many small, uniform square blocks {Block_*i*_} and then determine the central pixel **p**_*i*_ for each block Block_*i*_. For **p**_*i*_ each record its pixel value in all frames. Determine the computational interval *n*.
2. Compute the standard deviation *a*_*i*_ and determine whether to update the background using Eq (4).
3. Calculate parameters using Eq (12) and recover the background model using Eq (11).
4. Smooth the background using an adaptive local noise reduction filter.

In actual applications, the vehicle counting system focuses only on the road surface. We do not need to consider trees or buildings. Block-wise methods dramatically reduce the amount of calculations used in the background update process, and the standard deviation is the essential indicator of whether to perform such an update.

**Fig 2:**
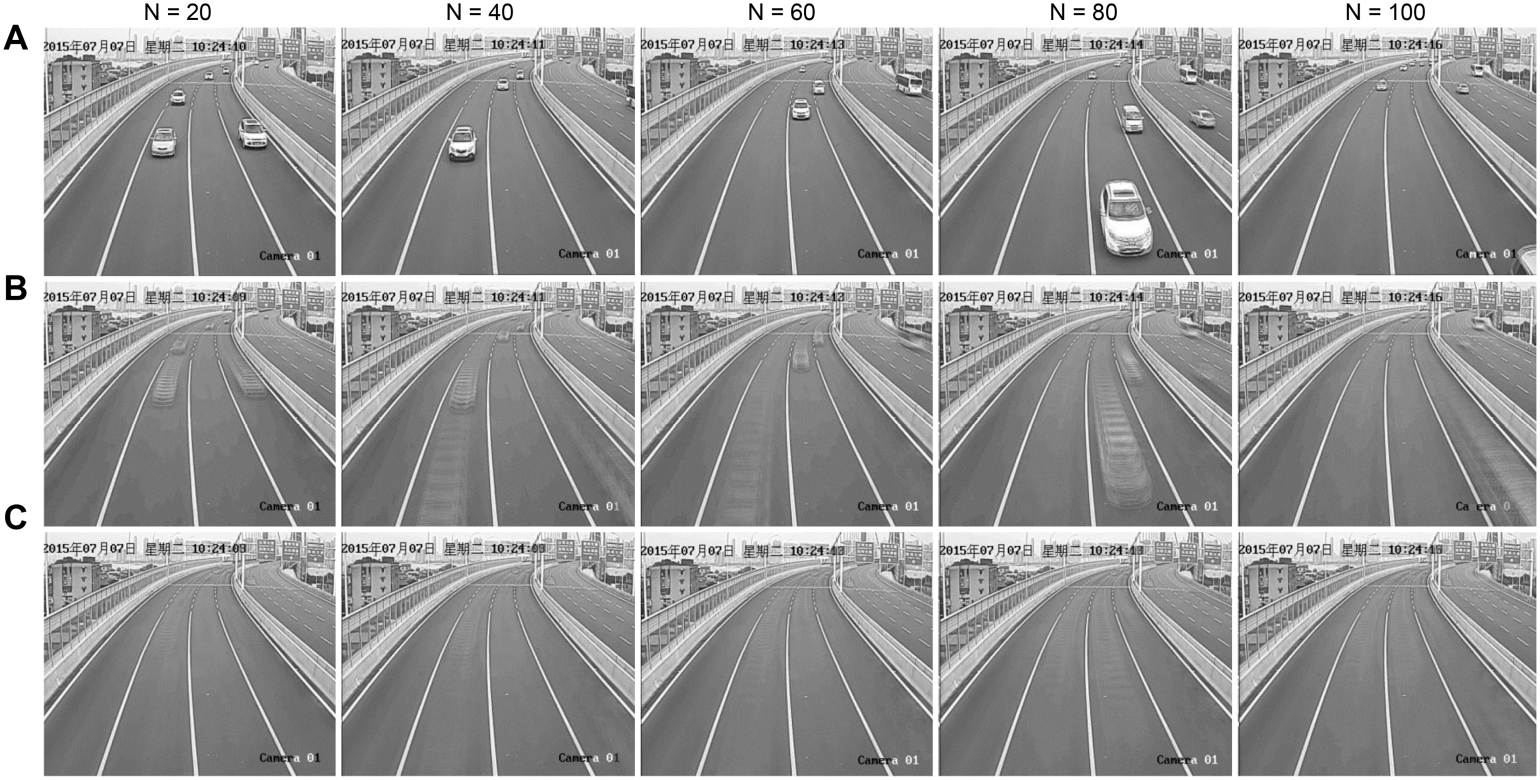
The background update result.

The result of background update is shown in Fig 2.

### Vehicle extraction

After the updated background image was obtained, the raw foreground image was subtracted from the grayscale image and was segmented to extract the real vehicles, which engages operations such as Otsu thresholding, shadow elimination, lamplight segmentation, and contour extraction. The shadows of vehicles will cause large detection errors in bright daylight. Shadow elimination is therefore an essential part of any vehicle detection algorithm. In this paper, a method proposed by Cucchiara et al. to accomplish shadow suppression based on hue, saturation, and value (HSV) color space information [17,18] is adopted. The method adopted in this paper to extract the vehicle contour is proposed by Suzuki [19].

## Traffic congestion detection

Existing vehicle detection and counting systems usually employ a simple algorithm for all traffic conditions, which makes it difficult to ensure the stability and robustness of the system. In this paper, the detection and counting proceed in different ways during congestion occasions and during free flow occasions. Therefore, we developed a novel detection method to monitor the real-time traffic state of the road section.

### Principle

**Fig 3:**
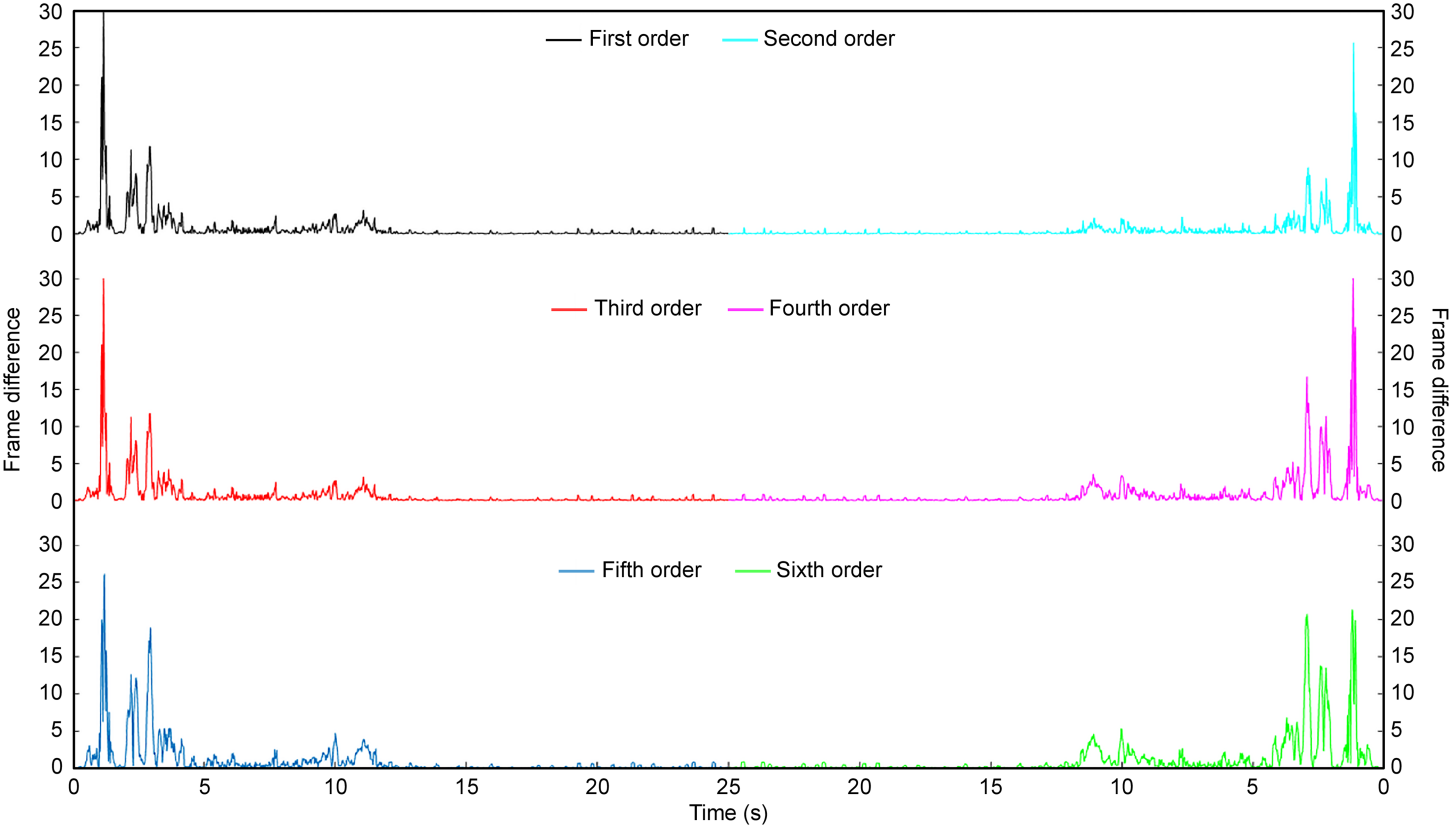
Frame difference function (FDF) of different orders.

Traffic congestion occurs when the traffic volume generates demand for more space than the available street capacity. Technically, a complicated complete model is needed for defining the traffic congestion state of a road. In this paper, traffic congestion is specifically defined to pave the way for counting slowly moving vehicles. For an urban road section, which is composed of serval lanes, traffic congestion of each lane can be modelled by assuming that vehicles in that lane move with sufficiently low speed within a certain period of time. As mentioned above, the standard deviation of the frame series could be a good indicator of traffic congestion, but its time response cannot meet the requirements of real-time traffic congestion detection. Thus, the standard deviation is not applicable as an indicator. However, it stands to reason that traffic congestion can be interpreted as a state in which little difference exists between adjacent frames, making the frame difference a potential criterion for identifying traffic congestion. In this paper, we propose a fast traffic congestion detection algorithm based on the frame difference function (FDF) and virtual loop. The experimental results perfectly support this idea, as shown in Fig 3.

**Fig 4:**
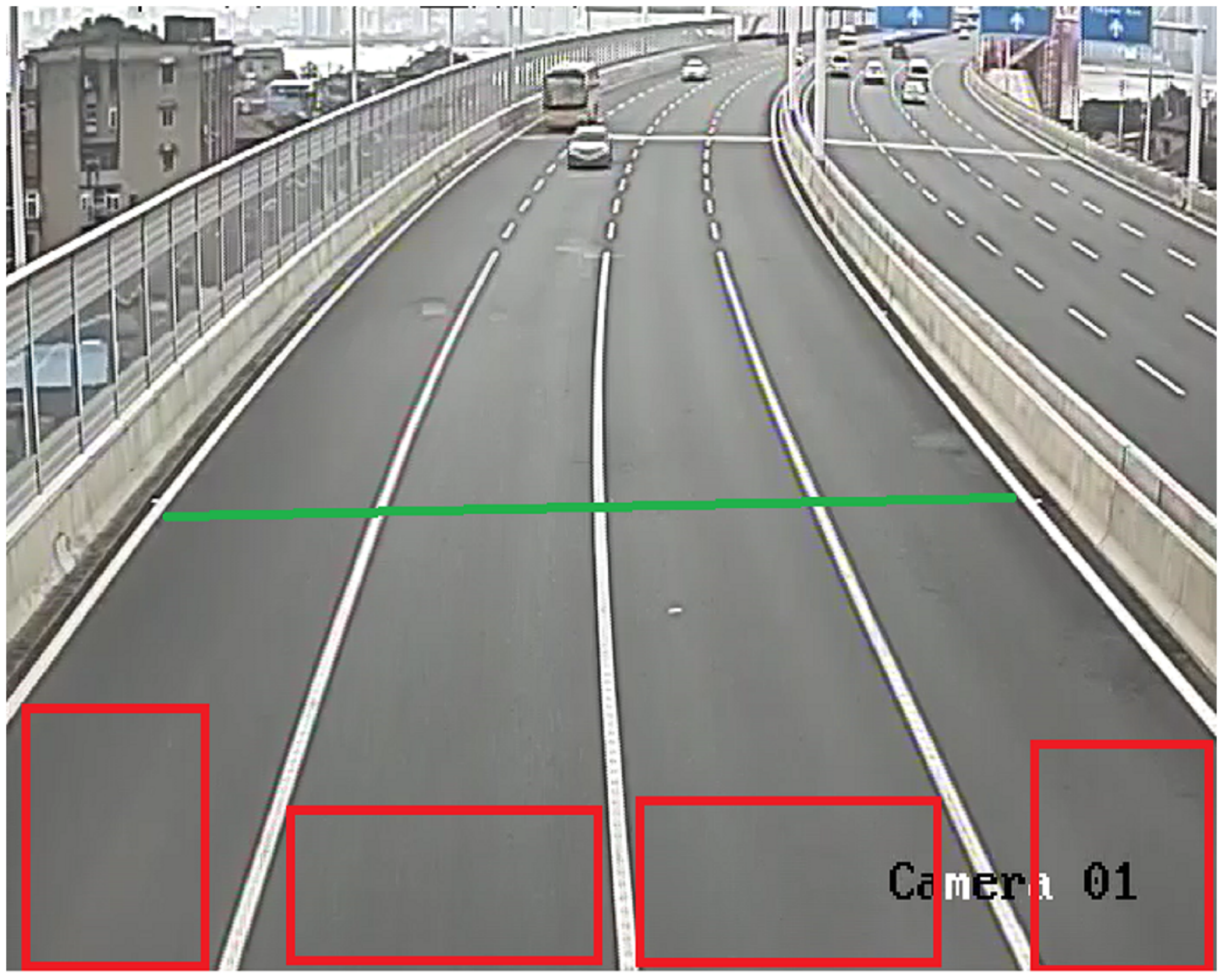
Virtual loops and the detection line.

The results of Fig 3 are obtained by calculating the difference between the average intensities of every 2, 3, 4, 5, 6, and 7 frames with respect to frame time in the region of a virtual loop (as shown in Fig 4). The source video is approximately 30 seconds long, starts with a normal traffic state and then ends with a traffic jam. As the video shows, three vehicles intrude in the area of the virtual loop; the first two leave immediately, but the first vehicle’s speed is much greater than that of the second. The third vehicle leaves approximately 5 seconds later. Another vehicle enters after one or two seconds, and then no vehicles move.

As shown in Fig 3, the vehicle’s movement produces an impulse-like change in the FDF in all orders as the period of traffic congestion corresponds to the flat area of the function. The response of a higher-order FDF to slow changes in the video is stronger than that of the first-order FDF. Thus, a higher order results in higher detail resolution. As the sixth graph shows, we can determine the exact time that a vehicle drives into or out of the virtual coil. The fourth- and sixth-order FDFs are priorities, and they have excellent performance when used in traffic congestion detection.

### Procedures

**Fig 5:**
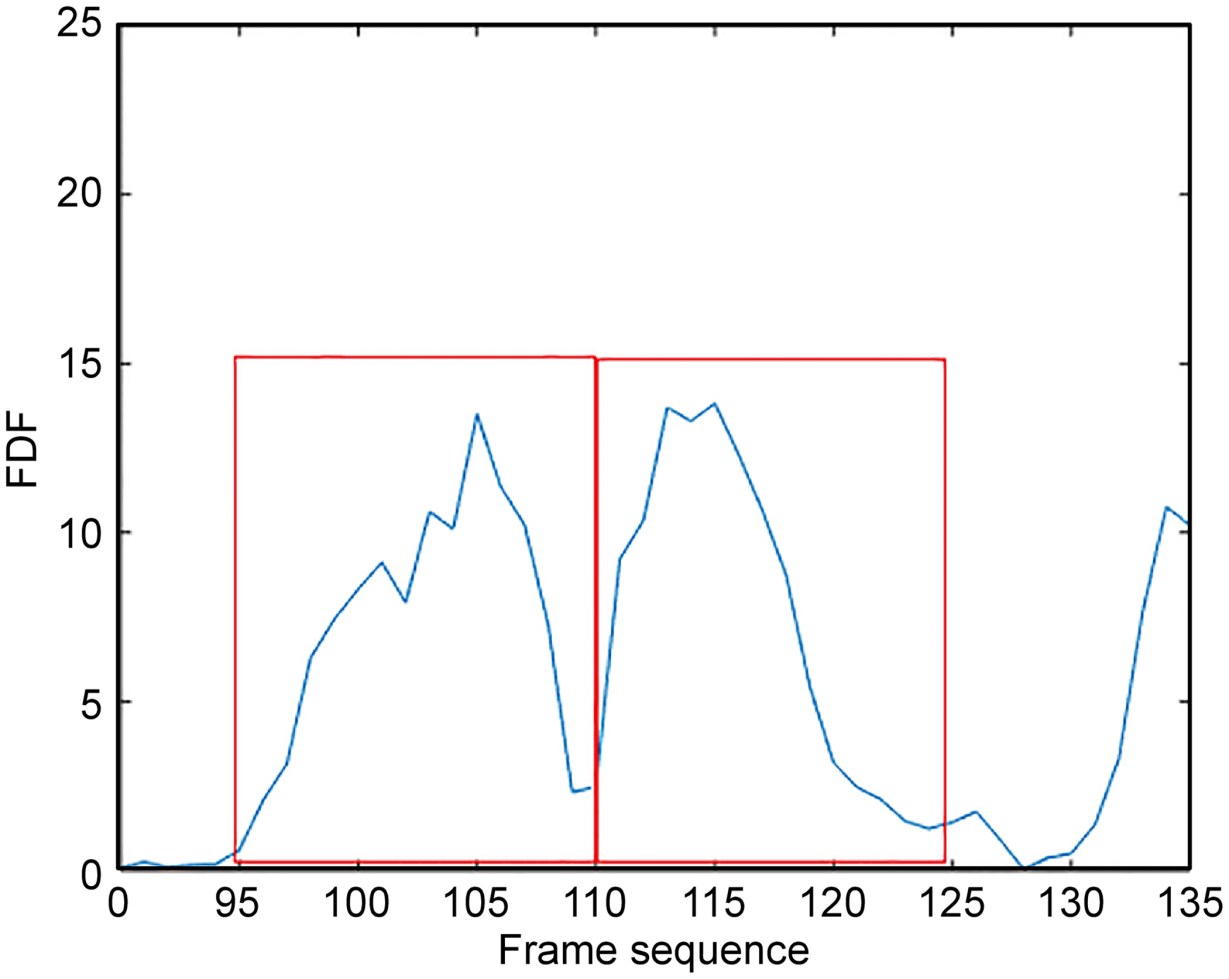
The sixth-order FDF with respect to frame sequence.

Several FDFs can be assembled together to detect the traffic congestion or to adjust to complicated scenarios. This study adopts the single sixth-order FDF for simplicity. The basic method is to construct a calculation window CW, as defined in section II, to identify the peaks of the FDF. The period should encompass at least the range of an impulse, which is approximately 15 frames long, as shown in Fig 5.

In this paper, every 20 consecutive frames form a calculation unit. Practical applications may require more calculation units, such as 2 units with a range of 40 frames, to produce a more precise traffic state prediction. The calculation window is determined as follows:

$${\text{CW}}_{\tau }=(num,aver,th3,th4,th5)$$ (14)

*num* is the count of FDFs whose values are larger than the threshold *th*5, and *aver* is the average FDF value of the calculation window. After completing the vehicle detection task, we can obtain the list of vehicles for every calculation frame, which is denoted by V. Thus, the traffic congestion status CW_τ_ can be obtained as follows:

$${\text{CW}}_{\tau }=\left\{ \begin{array}{l} normal,aver>th4\;\text{and}\;num>th3\;\text{and}\;\text{V}\neq null\\ clear,aver<th4\;\text{and}\;\text{V}=null\\ congested,aver<th4\;\text{and}\;\text{V}\neq null \end{array}\right.$$ (15)

The threshold values *th*3 and *th*4 ensure that only valid vehicle signals are considered.

The FDF calculation for each lane proceeds asynchronously; thus, the traffic states of the lanes may not be the same. However, the traffic state of the target road section must all be the same. Only when all the lanes are congested is the traffic state of the road section considered “*congested*”. The real-time traffic state of the road section is returned as reference data to the database.

## Vehicle counting

The existing video-based vehicle counting systems usually apply a single algorithm to count vehicles, such as setting baselines [20] or using virtual loops [21]. Methods based on line detection are suitable for counting vehicles with high speed. In traffic congestion, the vehicles are close to each other and move at a low speed; thus, there is a greater risk of counting two adjacent vehicles as one. Virtual loops are rectangles inside a single lane, they can be considered as an extension of a parallel line detection pair or as a simulation of an inductance loop. Since the whole area of the loop needs to be calculated, the computational time of this method is relatively high. However, the methods based on virtual loops can effectively perform counting in congested traffic. Based on the advantages of the two methods, we propose an adaptive counting algorithm that can automatically shift between the two patterns, using detection lines for normal traffic and virtual loops for congestion.

### Counting based on the detection line

#### Principles

The detection line is a custom virtual line that cuts through the road, as shown in Fig 4. The detection line should not be too far from the camera to maintain a certain distance between the virtual loops. The detection line should reach the two sides of a single lane road. A vehicle driving across the detection line will always intersect the line in the perspective of the image plane.

If a segment of the line is cut by a vehicle (represented by the circumscribed rectangle of the vehicle), we set the state of the segment as “*occupied*”. After the vehicle has deviated from the line, the segment is released, and its status is set to “*released*.” The vehicle count for the corresponding lane is then updated.

**Fig 6:**
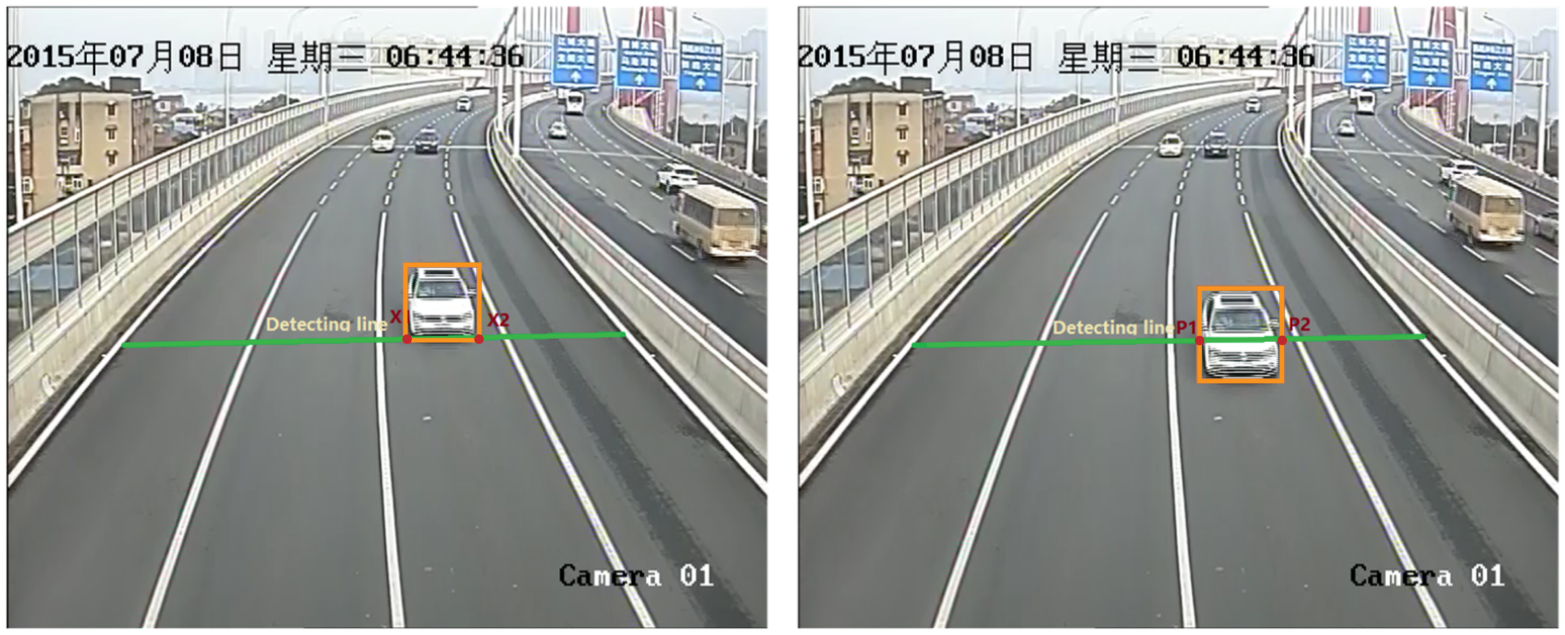
A special case in detection line.

The procedure is listed below:

**Step 1**: Obtain the four corner coordinates of the vehicle’s circumscribed rectangle. Determine whether the rectangle cuts the line based on a coordinate-wise comparison. If the line intersects the rectangle (as shown in Fig 6(a)), go to step 2; otherwise, go to step 3;
**Step 2**: Find the coordinates of the two intersection points, which form a segment belonging to the detection line. Set the status of the segment as *occupied*. Acquire the rectangle of the vehicle in the next frame, and then go to Step 1.
**Step 3**: Determine whether the rectangle is above or below the detection line. If the result is “*above*”, then do nothing; otherwise, obtain the projection of the rectangle on the detection line, and find its status. If the status of the projection segment is “*occupied*”, add 1 to the vehicle in the corresponding lane and release the segment; otherwise, acquire the next frame and go to Step 1.

#### Improvements

Not every vehicle has its apex vertical to the detection line, and the rectangles of the same vehicle in two different frames may not be the same. Thus, the occupancy of the vehicle rectangle on the detection line varies from time to time, which is shown in Fig 6(b). The vehicle drives through the detection line, and the segment that it occupies is X1X2. In a later frame, the segment it occupies changes to P1P2. In this state, the computer does not know if the vehicle is the previous one because its relevant segment in the detection line is not the same as before. Thus, the segment needs to be updated for every new frame.

Let *x*_1_, *x*_2_, *p*_1_ and p_2_ denote the X-coordinates of the points X1, X2, P1, and P2, respectively. Define the overlap ratio of the two segments *φ* as:

$$\varphi =\left\{ \begin{array}{l} (x_{2}-p_{1})/(x_{2}-x_{1}),\text{if}\;x_{1}\leq p_{1}\leq x_{2}\leq p_{2}\\ (p_{2}-x_{1})/(x_{2}-x_{1}),\text{if}\;p_{1}\leq x_{1}\leq p_{2}\leq x_{2}\\ 1,\text{if}\;p_{1}\leq x_{1}\leq x_{2}\leq p_{2}\\ (p_{2}-p_{1})/(x_{2}-x_{1}),\text{if}\;x_{1}\leq p_{1}\leq p_{2}\leq x_{2}\\ 0,\text{otherwise} \end{array}\right.$$ (16)

If *φ* exceeds 0.5, which means that the overlap ratio is greater than 50 percent, then the same car is driving through the “two” segments. Then, update the segment that the vehicle occupies to P1P2. If *φ*<0.5 another vehicle in another lane likely occupies segment P1P2.

In practical applications, errors exist between the estimated vehicle and the real vehicle in both location and size. The circumscribed rectangle does not intersect with the detection line when a vehicle leaves. Thus, the detection segment with the *occupied* status will not be released. To solve this problem, we consider more frames instead of using only the next frame, which improves the robustness of the algorithm and avoids repeated counting or underestimation.

### Counting based on virtual loops

The virtual loop method is a vehicle counting method similar to the inductance loop buried beneath the road surface. As Fig 5 shows, there is a virtual loop at the bottom center of every lane, and the length of the virtual loop is the same as that of the lane. This is an imaginary region that can be specified by customers.

Similar to the detection segment, every virtual loop has a status flag, denoted by S_*vc*_, that is defined as follows:

$${\text{S}}_{vc}=\left\{ \begin{array}{l} 1,\text{if}\;\text{the}\;\text{virtual}\;\text{loop}\;\text{is}\;\text{not}\;\text{empty}\\ 0,\text{otherwise} \end{array}\right.$$ (17)

The concrete method for determining the value of S_*vc*_ is to calculate the ratio of the object pixels to all pixels and the average width of the object in the loop.

First, we calculate the final binary image with the detected objects lying in the loop. We find the number of pixels, denoted by *M*, that belong to the object. Next, we assume that the size of the loop is *l* * *w*, where *l* and *w* are the length and the width of the loop and have units of pixels. Then, the object pixel ratio *δ* can be calculated using the equation below:

δ=M/(w*l) (18)

The object inside a loop is a connected region. The average width of the object, denoted as *W*, can be obtained via image processing tools. The ratio of the width of the object to the width of the loop *λ* is expressed as:

λ=w/l (19)

The experimental results show that S_*vc*_ can be summarized as follows:

$${\text{S}}_{vc}=\left\{ \begin{array}{l} 1,\text{if}\;\delta \geq 0.1\;\text{and}\;\lambda \geq 0.35\\ 0,\text{otherwise} \end{array}\right.$$ (20)

Let C_*i*_ denotes the current count of the *i*th lane, which is updated as follows:

$${\text{C}}_{i}=\left\{ \begin{array}{l} {\text{C}}_{i},{\text{S}}_{vc}:0\to 0\\ {\text{C}}_{i}+1,{\text{S}}_{vc}:0\to 1\\ {\text{C}}_{i},{\text{S}}_{vc}:1\to 0\\ {\text{C}}_{i},{\text{S}}_{vc}:1\to 1 \end{array}\right.$$ (21)

### Automatic shifting between the two counting patterns

Based on the traffic congestion detection method proposed in the previous chapter, the counting pattern of the system can automatically shift between the two methods with excellent speed performance. Assuming that the current traffic status is uncongested, the system will count the vehicles based on a single detection line. When a traffic jam happens in any of the three lanes, the counting pattern immediately shifts to the virtual loop method. Once the traffic congestion clears, all the patterns change to the normal state in a similar fashion.

## Results and discussion

### Vehicle counting

The accuracy of the vehicle counting in our system along with the results of some existing systems are listed in Table 1.

**Table 1:** Comparison with existing models.

Model	This paper	1	2	3	4	5	6	7
**Accuracy (%)**	99.29	90.17	89.8	87.78	94.17	96.4	94.04	97.4
**Max-Min (%)**	0.19	11.1	10.2	28.08	4.37	7.2	4.5	2.8

The results are obtained by calculating the average accuracy for different scenarios using systems listed as follows: 1: Chen et al. for car detection [20]; 2: Chen et al. [21]; 3: Lei et al. [22]; 4: Pornpanomchai et al. [23]; 5: Rodríguez and García [24]; 6: Mohana et al. [25]; 7: Li et al. [26]. The average accuracy of vehicle counting for our system reaches 99.29%, which surpasses that of all the other listed algorithms, which is not entirely unexpected. Table 1 also shows that the max-min difference among different occasions of our system remains at a very low level, which helps to determine the robustness of our system. On one hand, the proposed background update method, which focuses on the road ROI, can construct a more accurate background model in most vehicle detection scenarios than the other systems, which helps the system to more precisely identify vehicles. On the other hand, the adaptive shifting mechanism between the two counting methods makes the system more immune to counting errors. Some of the other auxiliary methods, such as shadow elimination and lamplight suppression, are employed to further improve the accuracy of vehicle counting.

To test the robustness of vehicle counting, 12 scenarios, including days and nights, rainy and sunny occasions, and congested and uncongested situations, are selected from the video records stored in DVR. The results are listed in Table 2.

**Table 2:** Results of vehicle counting.

Date	Period	Environment	Vehicle Counts		Error	Accuracy
			Actual	Estimated		
**2013/07/09**	**06:55~07:55**	Morning, congested^a^	2102	2082	0.78%	99.22%
	**14:51~15:51**	Day, normal^b^	1669	1656	0.80%	99.20%
	**16:50~17:50**	Day, normal	2122	2109	0.61%	99.39%
	**18:50~19:50**	Day, dusk, night, congested	1565	1556	0.58%	99.42%
	**19:51~20:51**	Night, normal	1144	1136	0.70%	99.30%
	**21:56~22:58**	Night, normal	1781	1764	0.95%	99.05%
	**22:59~23:59**	Night, normal	718	712	0.84%	99.16%
**2013/09/10**	**08:19~19:19**	Day, normal	2142	2126	0.75%	99.25%
	**13:21~14:18**	Day, rainy	1624	1619	0.31%	99.69%
	**20:08~21:04**	Night, rainy	1156	1150	0.52%	99.48%
	**21:56~22:58**	Night, normal	1781	1764	0.95%	99.05%

^**a**^ “congested” means that the traffic is congested in one or several time intervals in the corresponding testing period

^**b**^ “normal” means no rain and no traffic congestion

As can be inferred from Table 2, the estimated results are always superior to the actual ones in this test. On one hand, some vehicles are extremely close to each other and will be recognized as a single one when driving through the same virtual loop in congested traffic. On the other hand, when the traffic flows with an unexpectedly high speed, the system may fail to detect some rapidly moving vehicles. There are also occasions when the detection result is correct but the counting system fails to count the detected vehicles, which is the main reason that the estimated numbers are smaller than the actual ones. Although not presented here, many issues occur when the estimated number of vehicles exceeds the actual number.

The average accuracy values of different situations are listed in Table 3.

**Table 3:** Results of different scenarios.

Scenario	Day	Transition	Night	Rainy	Congested	Normal
**Accuracy (%)**	99.35	99.42	99.21	99.59	99.14	99.20

It is no surprise that the accuracy differences among various scenarios are quite small. As explained above, the system adopts an adaptive background updating algorithm, an adaptive counting pattern and a few auxiliary methods to ensure the accuracy of the estimated background model and of vehicle counting.

Overall, the counting accuracy in the daytime scenario is higher than that in the nighttime scenario, which coincides with the expectation. The uniformity of illumination in the daytime is higher than that in the nighttime. Thus, the risk of a vehicle being blended into the background at night is much higher than in the day. The smallest accuracy occurs for the congested scenario, as expected. During traffic congestion, vehicles are too close to each other, which may cause some counting errors. Interestingly, the counting accuracies of the rainy (light rain) scenarios are slightly higher than those of the normal condition. This is partially because the rain washes both the background and the vehicles, which has an image sharpening effect. The sharpening enhances the edges of the vehicles, making it easier to find the vehicle contours and improving the performance of the gradient operator used in lamplight suppression.

### Time performance of the system

The algorithm running time performance of several comparable algorithms are listed in Table 4. The results were obtained by running the program on a personal computer, which revealed a satisfactory output.

**Table 4:** Time performance of major algorithms computed in 1000 frames.

Function/Measure	Average/ms	Variance	Max/ms	Min/ms
**Detection**	58.78	5.2440	76	56
**Background Update**	5.72	2.0016	10	3
**Count**	2.83	1.6981	12	2

Although the fps of the video source can reach 25, experiments have shown that only one-third of the frames are needed to perform the calculations, which leaves the system 120 *ms* at most to process one frame. As shown in Table 4, the average detection time is 58.78 *ms*. The vehicle detection process includes background subtraction, threshold segmentation, contour extraction, smoothing, shadow removal, lamplight suppression and other necessary calculations, so it takes the most time. Lamplight usually happens at night, and shadows are usually produced during daylight, so the calculations for detection change significantly. Thus, the variance in the detection time is not small. In addition, the number of vehicles changes randomly from time to time, which also contributes to the large variance. Background update and vehicle counting use only typical pixel calculations, so they resolve with promising speed. The background update equation and ghost management are executed, although not in every frame, so the max time (10 *ms*) is much longer than the min time (3 *ms*, only the indicator is calculated). The time consumed during vehicle counting based on a detection line is much smaller than that used in vehicle counting based on the virtual loop, which explains the large difference between the max and min counting times.

The processing time is 67.33 *ms* on average and 98 *ms* in the worst-case condition, which are both smaller than the critical processing time of 120 *ms*. The real-time performance of the system when run on high-performance server computers will exceed the results above.

## Conclusions

Traffic flow information is extremely important in crowded modern cities. Because they are powerful, comprehensive, instantaneous, simple and cheap, video-based systems almost have no rivals in vehicle detection, counting and monitoring tasks. Based on state-of-the-art sequential image processing technologies, an adaptive real-time vehicle detection and counting system is proposed. Armed with a powerful adaptive real-time block-wise background updating algorithm, the system produces highly accurate detection results. Adopting an intelligent counting method, which cooperates with two different counting methods, helps improve the counting accuracy of the system. Other modified algorithms such as lamplight suppression, nighttime checking and shadow elimination also contribute to the resulting monolithic system that can operate with excellent performance. Finally, the traffic congestion detection algorithm serves as an effective indicator of the traffic status and could provide adequate congestion data for various applications. The results show that the detection and counting accuracy are satisfactorily high and that the system’s real-time performance meets or exceeds expectations. Nevertheless, every detail is worth improving in further research. In this paper, we have not tested the system in heavy weather conditions as real-time vehicle detection and counting in heavy weather conditions such as heavy fog or haze, heavy rain or snow remains a difficult topic in computer vision area which propose big challenges in our further research. A series of methods that detect and deal with all kinds of heavy weathers should be integrated into the system to help improve its robustness. Above all, the system we developed is validated as efficient and robust.

## Supporting information

S1 File The impacts of foggy weather on vehicle counting. (DOCX)
