# Supplementary material for: A video-based real-time adaptive vehicle-counting system for urban roads
Source: PLoS One. 2017 Nov 14;12(11):e0186098. doi: 10.1371/journal.pone.0186098 (PMC5685594; doi:10.1371/journal.pone.0186098)
Supplement: S1 File — (DOCX) [file pone.0186098.s001.docx]

# The impacts of foggy weather on vehicle counting

## Results and discussion

Technically, fog or haze occasions have lighter impacts on images than that of the rainy occasions. Fog can be recognized as a kind of uniformed noise to images which has little impacts on the contours of the objects. While kernel task of vehicle counting system is to detect the contours of moving vehicles. Besides, light fog or haze can be easily removed with using simple sharpening filters or some advanced filters. The counting accuracy in light fog or haze occasions should be better than that of the rainy weather. Therefore, fog or haze weather conditions are not taken into account in this paper. To prove this point, we did experiments in some fog conditions. It has to be mentioned that both light fog or dense fog in this paper are not sufficient to cause irreversible damage to the images. The results of vehicle counting under light fog condition and dense fog conditions are shown in Table 1 and Table 2. The de-fog algorithm we developed is based on dark channel technology [1-4].

**Table 1. Results of vehicle counting in light fog occasion**

| **Actual vehicles** | **Light fog^*^** | **Error** | **After de-fog** | **Error** |
| --- | --- | --- | --- | --- |
| 200 | 201 | 0.50% | 201 | 0.50% |
| 400 | 403 | 0.75% | 403 | 0.75% |
| 600 | 603 | 0.50% | 603 | 0.50% |
| 800 | 805 | 0.63% | 805 | 0.63% |
| 1000 | 1004 | 0.40% | 1004 | 0.40% |

*Visibility range from 1000m to10000m

**Table 2. Results of vehicle counting in dense fog occasion**

| **Actual vehicles** | **dense fog^*^** | **Error** | **After de-fog** | **Error** |
| --- | --- | --- | --- | --- |
| 200 | 199 | 0.50% | 200 | 0% |
| 400 | 397 | 0.75% | 401 | 0.25% |
| 600 | 595 | 0.83% | 601 | 0.13% |
| 800 | 795 | 0.63% | 802 | 0.25% |
| 1000 | 994 | 0.60% | 1002 | 0.20% |

*Visibility range from 50m and 500m

Under normal weather condition, the counting accuracy of the system is 99.2%. As we can see from Table 1 and Table 2, vehicle counting accuracy in light fog occasion and dense fog occasion ranges from 99.17% to 99.6%. Comparing the counting results of fog occasion and after de-fog occasion we can see that light fog hardly lower the accuracy. Dense fog causes a bigger error in counting accuracy when the count of vehicle grows than light fog while the error still remains at a very low level (<0.6%) . Therefore, fog has few impacts on vehicle counting accuracy. That is the one of the reasons that we exclude fog or haze weather from normal testing.

Table 1 shows that de-fog algorithm can hardly improve the counting accuracy. Table 2 shows that the de-fog algorithm helps reduce the counting error by nearly 0.4%, which is a promising result. However, dark-channel-prior-based haze removing algorithm runs slowly and is not applicable to real-time vehicle counting. It’s our plan to develop a fast and efficient de-fog algorithm in our further researches.

## Reference

1. He K, Sun J, Tang X. Single image haze removal using dark channel prior[C]// Computer Vision and Pattern Recognition, 2009. CVPR 2009. IEEE Conference on. IEEE, 2009:1956-1963.
2. Ancuti C O, Ancuti C. Single Image Dehazing by Multi-Scale Fusion[J]. IEEE Transactions on Image Processing, 2013, 22(8):3271.
3. He K, Sun J, Tang X. Guided Image Filtering[J]. IEEE Transactions on Pattern Analysis & Machine Intelligence, 2013, 35(6):1397.
4. Hautiére N, Tarel_Affn J P, Lavenant_Affn J, et al. Automatic fog detection and estimation of visibility distance through use of an onboard camera[J]. Machine Vision & Applications, 2006, 17(1):8-20.
